# Oxytocin efficacy is modulated by dosage and oxytocin receptor genotype in young adults with high-functioning autism: a 24-week randomized clinical trial

**DOI:** 10.1038/tp.2016.152

**Published:** 2016-08-23

**Authors:** H Kosaka, Y Okamoto, T Munesue, H Yamasue, K Inohara, T Fujioka, T Anme, M Orisaka, M Ishitobi, M Jung, T X Fujisawa, S Tanaka, S Arai, M Asano, D N Saito, N Sadato, A Tomoda, M Omori, M Sato, H Okazawa, H Higashida, Y Wada

**Affiliations:** 1 Research Center for Child Mental Development, University of Fukui, Eiheiji, Japan; 2 Department of Neuropsychiatry, Faculty of Medical Sciences, University of Fukui, Eiheiji, Japan; 3 Division of Developmental Higher Brain Functions, Department of Child Development United Graduate School of Child Development, Osaka University, Kanazawa University, Hamamatsu University School of Medicine, Chiba University and University of Fukui, Eiheiji, Japan; 4 Research Center for Child Mental Development, Kanazawa University, Kanazawa, Japan; 5 Department of Neuropsychiatry, School of Medicine, University of Tokyo, Tokyo, Japan; 6 Department of Informatics, Graduate School of Informatics and Engineering, The University of Electro-Communications, Chofu, Japan; 7 International Community Care and Lifespan Development, Empowerment Sciences, Faculty of Medicine, University of Tsukuba, Tsukuba, Japan; 8 Department of Obstetrics and Gynecology, University of Fukui, Eiheiji, Japan; 9 Department of Child and Adolescent Mental Health, National Institute of Mental Health, National Center of Neurology and Psychiatry, Kodaira, Japan; 10 Biomedical Imaging Research Center, University of Fukui, Eiheiji, Japan; 11 Department of Cerebral Research, National Institute for Physiological Sciences, Okazaki, Japan; 12 Faculty of Nursing and Social Welfare Sciences, Fukui Prefectural University, Eiheiji, Japan; 13 Department of Anatomy and Neuroscience, Graduate School of Medicine, Osaka University, Suita, Japan; 14 Division of Developmental Neuroscience, Department of Child Development United Graduate School of Child Development, Osaka University, Kanazawa University, Hamamatsu University School of Medicine, Chiba University and University of Fukui, Suita, Japan

## Abstract

Recent studies have suggested that long-term oxytocin administration can alleviate the symptoms of autism spectrum disorder (ASD); however, factors influencing its efficacy are still unclear. We conducted a single-center phase 2, pilot, randomized, double-blind, placebo-controlled, parallel-group, clinical trial in young adults with high-functioning ASD, to determine whether oxytocin dosage and genetic background of the oxytocin receptor affects oxytocin efficacy. This trial consisted of double-blind (12 weeks), open-label (12 weeks) and follow-up phases (8 weeks). To examine dose dependency, 60 participants were randomly assigned to high-dose (32 IU per day) or low-dose intranasal oxytocin (16 IU per day), or placebo groups during the double-blind phase. Next, we measured single-nucleotide polymorphisms (SNPs) in the oxytocin receptor gene (*OXTR*). In the intention-to-treat population, no outcomes were improved after oxytocin administration. However, in male participants, Clinical Global Impression-Improvement (CGI-I) scores in the high-dose group, but not the low-dose group, were significantly higher than in the placebo group. Furthermore, we examined whether oxytocin efficacy, reflected in the CGI-I scores, is influenced by estimated daily dosage and *OXTR* polymorphisms in male participants. We found that >21 IU per day oxytocin was more effective than ⩽21 IU per day, and that a SNP in *OXTR* (rs6791619) predicted CGI-I scores for ⩽21 IU per day oxytocin treatment. No severe adverse events occurred. These results suggest that efficacy of long-term oxytocin administration in young men with high-functioning ASD depends on the oxytocin dosage and genetic background of the oxytocin receptor, which contributes to the effectiveness of oxytocin treatment of ASD.

## Introduction

Autism spectrum disorder (ASD) is characterized by persistent deficits in social communication and social interaction across multiple contexts and restricted, repetitive patterns of behavior, interests, or activities.^1^ Although no pharmacologic treatments exist for the core symptoms of ASD, recent studies have proposed that intranasal oxytocin administration may be effective.^2, 3^

Recent randomized controlled trials (RCTs) reported positive effects of long-term oxytocin administration in adults with ASD,^4, 5, 6^ although pediatric efficacy remains controversial.^7, 8, 9^ Anagnostou *et al.*^4^ found that performance on the Reading the Mind in the Eyes Task (RMET) and Quality of Life Questionnaire improved after 6-week administration of 48 IU per day of oxytocin in adults with ASD.^4^ Watanabe *et al.*^5^ found that 6-week administration of 48 IU per day oxytocin improves Autism Diagnostic Observation Schedule reciprocity scores and brain activity in adults with ASD.^5^ In male adolescents and adults with ASD and comorbid intellectual disabilities, reciprocal social interactions observed during play sessions or in daily life significantly increased after 8-week administration of 16 IU per day.^6^ These findings suggest that long-term oxytocin administration can alleviate core symptoms in adults with ASD.

However, several issues need to be resolved before clinically using oxytocin treatment for adults with ASD.^3, 10, 11^ For instance, it has been proposed that the efficacy of oxytocin administration depends on dosage.^10, 12, 13^ Previous animal^14, 15^ and human studies on diseases other than ASD^16, 17, 18^ have shown dose-dependent oxytocin efficacy. A clinical study with divergent single-dose oxytocin administration in patients with schizophrenia showed that 20 IU oxytocin, but not 10 IU oxytocin, ameliorated emotion recognition deficits in a polydipsic group.^18^ However, as the effect of oxytocin administration differs between schizophrenia and ASD,^19^ dose dependency of oxytocin administration in individuals with ASD requires investigation. Nevertheless, no trial has examined the dose-dependent efficacy in individuals with ASD by manipulating daily dosage.

Furthermore, the genetic background of oxytocin system might influence efficacy of oxytocin administration.^19, 20^ Intranasally administered oxytocin is considered to act through the oxytocin receptor (*OXTR*).^21, 22^ As *OXTR* contains several dozen single-nucleotide polymorphisms (SNPs), efficacy of long-term administration might differ according to *OXTR* gene polymorphisms. A single-dose study in healthy volunteers showed that *OXTR* gene polymorphisms altered sensitivity to reward-relevant features of infants and/or their aversive properties^20^ and that improvement of neural response associated with social cooperation differs by *OXTR* gene polymorphisms.^23^ Previous studies reported that genetic alteration of *OXTR* is associated with ASD occurrence;^21, 24^ therefore, association of genetic polymorphisms with responsiveness to oxytocin might differ between individuals with and without ASD. Nonetheless, the association between efficacy of long-term oxytocin administration and SNPs in *OXTR* among individuals with ASD remains unclear.

In this trial, to examine whether dosage and *OXTR* gene polymorphisms affect efficacy of oxytocin administration, we performed a phase 2 clinical pilot trial with 12-week intranasal oxytocin administration in young adults with high-functioning ASD. We set two different dosage group (32 and 16 IU per day) and measured SNPs in *OXTR*, to examine whether estimated daily dosage amount and SNPs in *OXTR* predict improvement of outcomes.

## Materials and methods

### Participants

We recruited participants from the University of Fukui Hospital, Kanazawa University Hospital and a few nearby clinics specializing in ASD treatment. Eligible participants were young male and female adults over 15 years of age diagnosed with autistic disorder or pervasive developmental disorder not otherwise specified as defined by the Diagnostic and Statistical Manual of Mental Disorders (DSM-IV-TR).^25^ Two psychiatrists (HK and TM) confirmed the diagnosis using the Diagnostic Interview for Social and Communication Disorders.^26^ We excluded participants with a history of major medical or neurological illnesses (that is, epilepsy, significant head trauma, a lifetime history of alcohol or drug dependence), those with syndromic forms of autism (for example, Rett syndrome and tuberous sclerosis), intellectual disabilities or other severe neuropsychiatric diseases (for example, schizophrenia, anxiety and mood disorders) and pregnant women. This clinical trial was approved by the institutional review board of the University of Fukui, Japan, and was conducted in accordance with the Declaration of Helsinki and the Ethical Guidelines for Clinical Studies of the Ministry of Health, Labour and Welfare of Japan. Written informed consent was obtained from each participant and parents if the participant was under 20 years old, after a complete explanation of the trial. For female participants, we obtained informed consent regarding the need for contraception and risks of long-term oxytocin administration.

### Procedure

Between March 2011 and March 2014, we conducted a single-center phase 2, pilot, randomized, double-blind, placebo-controlled, parallel-group, clinical trial at the Department of Neuropsychiatry of University of Fukui Hospital, Japan (UMIN000005211). The trial duration was 32 weeks, including a 12-week double-blind phase, 12-week open-label phase and 8-week follow-up phase. Dose-dependent efficacy of oxytocin administration was assessed in the double-blind phase, and safety was monitored in all phases. Our open-label study showed that Clinical Global Impression-Improvement (CGI-I) score gradually improved over 6 months.^27^ Therefore, we set a longer evaluation period (12 weeks) than that in the previous RCT^4, 5, 6, 7, 8, 9^ to increase the efficacy. Participants were randomly assigned (1:1:1) to high-dose oxytocin, low-dose oxytocin and placebo groups during the double-blind phase. We recruited 20 participants with ASD for each group regardless of sex. Therefore, a total of 60 individuals with ASD in three groups participated in this pilot trial. The randomization schedule was generated by an unmasked statistician not involved in conducting the trial and data analysis. Randomization was centralized, using a computer-generated list with random block sizes of six. Participants, their family and staff were masked to the treatment, and the allocation sequence was not disclosed until the open-label phase for all the participants was completed. During the double-blind phase, participants received 32 IU per day (high-dose group) or 16 IU per day oxytocin (low-dose group), or placebo, in the form of an intranasal spray. The active drug, Syntocinon spray (Novartis, Basel, Switzerland), was transferred from the original bottle to a sterile nasal spray bottle (Oono, Tokyo, Japan). In the open-label phase, all participants received the same oxytocin dose (32 IU per day). To control the usage of the spray, we provided detailed instructions on how to use the spray and confirmed the participant's usage at every visit. Each participant visited the hospital every 4 weeks during the trial.

### Efficacy and safety assessments

The primary outcomes were the CGI^28^ and Interaction Rating Scale Advanced (IRSA)^29^ scores. The CGI-Severity (CGI-S), which evaluates ASD severity on a 7-point scale (from 1 ‘normal, not at all ill' to 7 ‘extremely ill'), was assessed by an expert psychiatrist (HK) at weeks 0, 12 and 24. The CGI-I, which evaluates the efficacy of oxytocin administration from week 0 on a 7-point scale (from 1 ‘very much improved' to 7 ‘very much worse'), was assessed by the same psychiatrist at weeks 12 and 24. To rate the CGI, the psychiatrist comprehensively assessed the participants' ASD core symptoms and comorbid symptoms in daily life including social behaviors at episodic event, based on total clinical experience. Videotaped interactions between each participant and the psychiatrist at weeks 0, 12 and 24 were assessed using the IRSA, which is designed to evaluate various communicative function such as assertiveness or responsiveness in adult–adult interactions.^29^

Oxytocin administration improves various psychiatric symptoms such as depression and anxiety, as well as social-communicative dysfunction.^30^ Therefore, we measured various psychiatric symptoms at weeks 0, 12 and 24 as a secondary outcome using self-report questionnaires of the Zung Self-rating Depression Scale, state anxiety scale of the State-Trait Anxiety Inventory, 20-item Toronto Alexithymia Scale and a parent-report questionnaire of the Aberrant Behavior Checklist.

For a subset of participants, we also measured gaze pattern using an eye-tracking system and brain activity using resting-state functional MRI at weeks 0 and 12 because changes of gaze behavior and brain function might result in improvement of core symptom or comorbidities (Supplementary Information).

Safety and tolerability were assessed by adverse events and vital signs. We measured body weight, body temperature, blood pressure and pulse rate at every visit. Blood examination including blood count, renal and liver function, thyroid and sex hormone levels (plasma testosterone, estradiol, progesterone, luteinizing hormones and follicle stimulating hormones), and plasma oxytocin level were evaluated at weeks 0, 12 and 24. Plasma oxytocin levels were quantified using a commercial oxytocin ELISA kit without extraction (Enzo Life Sciences, Farmingdale, NY, USA).^31^ Specifically, we focused on the safety of female participants by having a gynecologist (MOr) monitor their sex hormone levels, menstrual cycle, and uterine peristalsis activity using cine MRI^32^ at weeks 0 and 24.

The acute effects of intranasal oxytocin are reported to persist until 60–80 min post-administration.^33, 34^ Therefore, we evaluated all outcomes >3 h after oxytocin administration in order to study the long-term rather than acute treatment effects of oxytocin.

### Single-nucleotide polymorphism of *OXTR*

To determine whether genetic disposition alters the efficacy of oxytocin administration, we identified SNPs in the *OXTR* (HGNC:8529) gene for each participant. We selected 24 SNPs based on the genotype data in the Japanese population from the HapMap Project^35, 36^ and previous studies examining their association with ASD^21, 24, 37^ (Supplementary Table 1). Genomic DNA was extracted from peripheral blood using standard phenol–chloroform methods with the QIAmp DNA Micro Kit (QIAGEN, Tokyo, Japan), and all SNPs were genotyped by real-time PCR analysis using TaqMan genotyping platform and StepOnePlus (Applied Biosystems, Foster City, CA, USA).

### Statistical analyses

**Figure 1:**
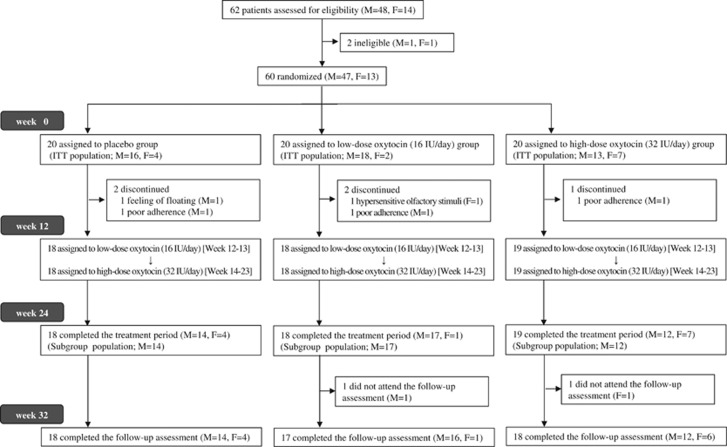
Trial profile. ITT population: intention-to-treat population, which included all study participants; Subgroup population: male participants with good adherence (>50%). F, female participants; ITT, intention-to-treat; M, male participants.

We conducted intention-to-treat (ITT) analyses including all participants, and subgroup analyses in only male participants with good adherence (>50%) because sex-based differences in responses to oxytocin administration have been reported (Table 1, Figure 1).^2, 38^ Statistical analyses were performed using IBM SPSS version 21 (IBM, Armonk, NY, USA) and R package ‘PARTY'.^39^

Initially, we examined oxytocin effects in the high-dose and low-dose groups during the double-blind phase. Because high-dose, but not low-dose, oxytocin administration improved the symptoms of schizophrenic patients,^18^ we hypothesized that the high-dose group shows prominent improvement. On the basis of this hypothesis, we utilized two-step analysis with a gatekeeping strategy^40^ to control the family-wise type 1 error rate. In the first step, we examined whether the high-dose group showed significant improvement in CGI-I score over the placebo group by independent *t*-test (two-sided) at week 12, and two-way analysis of variance (ANOVA) with groups (high-dose/placebo) and time (weeks 0/12) for the other outcomes. In the second step, we examined whether low-dose group shows significant improvement over the placebo group, but only for outcomes that showed significant improvement in the first step of analysis. Correction of multiple comparisons was conducted with two-sided *α=*0.004 (0.05/11, number of primary and secondary outcomes). To understand the size of this effect, we also evaluated Cohen's effect size.^41^

Subsequently, among outcomes that revealed significant improvement by oxytocin administration, we examined whether efficacy is influenced by dosage and *OXTR* gene polymorphisms in the two oxytocin groups. We utilized random forest regression with conditional inference trees (CTree),^42, 43^ which is a type of machine learning that is widely used. Random forests are a recursive partitioning method particularly well-suited to small *n* large *p* problems.^44^ In order to precisely evaluate dosage amount, the estimated daily oxytocin dosage was calculated by averaging the total residual quantity of the entire spray bottle for the 84-day double-blind period for each participant. Furthermore, within the 24 SNPs, SNPs which deviated from the Hardy–Weinberg equilibrium in the data set (*P*<0.05) or had minor-allele frequencies below 5% in the Japanese population, were excluded from the analysis. We set the estimated daily oxytocin dosage and genotype of each SNP scored 0, 1 or 2 as predictor values, and outcome was set as the dependent value.

## Results

### Participants and trial profile

We enrolled 62 eligible Japanese participants between 18 March 2011 and 24 September 2013, and two of them were excluded for not meeting diagnosis criteria (Figure 1). Therefore, 60 participants (47 males and 13 females, aged 15–39 years (mean 24.2 years), full-scale IQ 76–133 (mean 100.0)) were randomly allocated to three groups (Table 1). Twenty-two participants (36.7%) had received psychotropic medications because of comorbid symptoms, with stable doses over a month prior to randomization.

During the double-blind phase, two participants discontinued the trial, and three participants with poor adherence (⩽50%), confirmed by self-report and residual quantity of the spray bottle, were excluded from the subgroup analysis (Figure 1). Therefore, 55 of 60 participants (91.7%) continued the intranasal spray administration throughout the trial period. We analyzed the 60 and 43 participants for ITT population and subgroup population (male participants with good adherence), respectively.

### Outcomes and safety

Initially, we examined oxytocin effects in high-dose and low-dose groups during the double-blind phase. In the first step of analysis (high-dose vs placebo group), *t*-test did not reveal a significant difference in CGI-I score and two-way ANOVA on the other outcomes revealed no significant interaction between group and time in the ITT population (Table 2). When we analyzed the subgroup of male participants, *t*-test revealed significant improvement in CGI-I score in the high-dose oxytocin group over the placebo group (*t*(24)=3.714, *P*=0.001, two-sided; Cohen's *d*=1.52) with high *post hoc* power (0.944). By contrast, two-way ANOVA of the other outcomes with group and time as factors revealed no significant interaction in the subgroup population (Table 2). In the second step of analysis (low-dose vs placebo group), *t*-test did not reveal a significant difference in CGI-I score between the two groups (*P*=0.08, two-sided). Collectively, these results indicate that male participants in the high-dose group, but not the low-dose group, showed significant improvement in CGI-I score, suggesting that dose-dependent efficacy exist. There was a significant correlation between the CGI-I score at week 12 and the estimated daily oxytocin dosage (*n*=43, *r*=−0.512, *P*<0.001).

**Figure 2:**
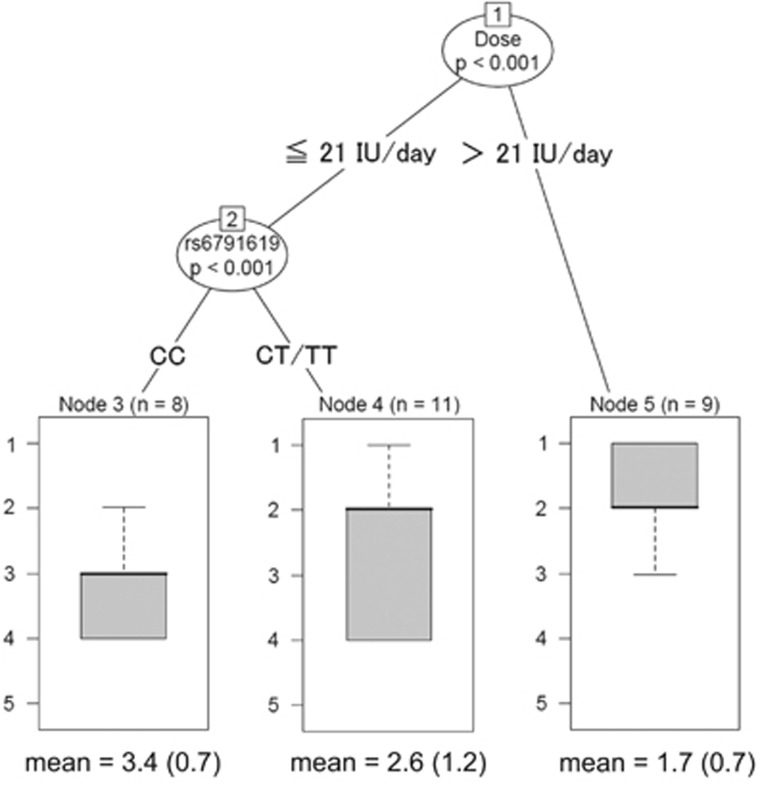
Assessment of the relative significance of variables (the estimated daily oxytocin dosage and 19 SNPs) for improvement of symptoms using conditional regression trees in the program package ‘PARTY' (implemented in R) for the subgroup population. The thick line shows the median values of the CGI-I score and the vertical dashed line shows the range. The lowest data are mean (s.d.) of the CGI-I score at week 12. CGI-I, Clinical Global Impression-Improvement; SNP, single-nucleotide polymorphism.

Subsequently, we examined whether efficacy was influenced by dosage and *OXTR* gene polymorphisms by analyzing CGI-I scores of the subgroup population at week 12 in both high-dose (12 males) and low-dose groups (16 males, excluding a male who refused to provide a blood sample). Estimated daily oxytocin dosages were 28.8±4.5 (18.9–32.0) and 13.5±1.4 IU (10.4–15.2) for the high-dose and low-dose groups, respectively. We excluded five SNPs (rs2270465, rs2301261, rs2268494, rs1042778 and rs237884) based on the exclusion criteria, and the remaining 19 SNPs were subjected to random forest regression analysis (Supplementary Table 1). As shown at the top of the tree in Figure 2, we estimated the daily oxytocin dosage as a superior predictor of CGI-I score compared with 19 SNPs in *OXTR* (Figure 2; *C*=6.59, *P*<0.001). As a second candidate, rs6791619 was also significantly predictive under lower-dose oxytocin treatment (⩽21 IU; Figure 2; *C*=5.42, *P*<0.001). Other SNPs were not significantly predictive of CGI-I score improvement. Collectively, participants receiving higher-dose (>21 IU) oxytocin showed stronger improvement of CGI-I score. Furthermore, when the dosage was lower (⩽21 IU), participants with a T-allele at rs6791619 showed stronger improvement.

We further examined whether gaze abnormality and altered brain function are affected by oxytocin administration for a subset of participants (Supplementary Information). We found that long-term administration of high-dose oxytocin has a tendency to increase gaze fixation on regions of social salience such as the eye region of the face and biological motion, with large effect size. In resting-state functional MRI analysis, we could not compare activation between high-dose and placebo groups because of the small number of participants in the high-dose group (*n*=1).

Across the clinical trial, no severe adverse events or abnormal physiological changes were observed (Table 3). Female participants did not complain about menstrual disorders and galactorrhea, and a gynecologist confirmed that there was no abnormality in sex hormone levels, menstrual cycle and uterine peristaltic activity. Two-way ANOVA on plasma oxytocin level revealed no main effects of group and time or interaction between group and time (*P*>0.05; Table 2).

## Discussion

In the pilot clinical trial, we confirmed that high-dose oxytocin administration for 12 weeks alleviates symptoms in male young adults with ASD with no severe adverse event. Furthermore, we provide novel evidence that the efficacy of oxytocin administration in male participants can be predicted by dosage and a SNP in *OXTR* (rs6791619).

### Efficacy of long-term oxytocin administration

In this trial, significant improvement was observed in CGI-I score but not in other outcomes for male participants in the high-dose group. To assess the CGI score, we comprehensively assessed the participant's ASD core symptoms and comorbid symptoms in daily life including social behaviors at episodic event, and showed that oxytocin administration can alleviate comprehensive symptoms. Previous findings also suggested that social-communicative skills, that is, Autism Diagnostic Observation Schedule reciprocity score^5^ and social interactions during the play sessions and daily life,^6^ were improved by long-term oxytocin administration;^5, 6^ however, we could not observe significant improvement in IRSA, which helps evaluate various social-communicate behaviors during medical interview. The inconsistent results might be because of difference in the assessment procedure. Autism Diagnostic Observation Schedule and social interactions measured by Munesue *et al.*^6^ were assessed by the evaluator after interacting with the participants. In contrast, the IRSA was assessed by videotaped interactions by the evaluator without interacting with the participants. Previous studies have found that oxytocin administration improves eye contact during naturalistic social interaction.^5, 45^ We also observed an increasing tendency to look at the eye-region for some face movies (that is, still face, blinking) with large effect size (Supplementary Information) in the high-dose oxytocin group. Such changes in gaze-behaviors are easier to detect by the person interacting with the participants than by a third person. Therefore, we could not detect changes in social-communicative behaviors by IRSA possibly because the assessment procedure used a videotaped interaction, in contrast to our findings of CGI-I score and those of previous trials.^5, 6^ Other than comprehensive symptom and social-communicative behaviors, we failed to find significant improvement of secondary outcomes assessing stereotypic behavior and comorbidity including anxiety and depressive state, which was consistent with the results of previous studies.^3, 4, 5, 6, 7, 8, 9^ Therefore, long-term administration of oxytocin might not be effective for improving stereotypic and repetitive behavior, and comorbid symptoms. Overall, our results show that comprehensive symptoms of males with ASD improved after administration of 32 IU oxytocin for 12 weeks.

### Efficacy of oxytocin administration is predicted by dosage and *OXTR* gene polymorphisms

In the trial, we provided novel evidence that administration of >21 IU per day oxytocin for 12 weeks is more effective than ⩽21 IU per day in treating comprehensive symptoms as measured by CGI-I score (Figure 2), which is consistent with findings of previous clinical trial in patients with schizophrenia.^18^ It is estimated that only a small amount of intranasally administered oxytocin reaches the brain.^33, 34, 46, 47^ For instance, a recent study showed that single-dose intranasal administration of 24 IU oxytocin increases the cerebrospinal fluid oxytocin level 1.5-fold in volunteers without ASD.^34^ Leng and Ludwig^48^ reported that a maximum of 0.005% of intranasally injected oxytocin reaches the cerebrospinal fluid within 1 h.^48^ This is likely because of the limited number of routes to reach the brain, such as blood-brain barrier and extraneuronal/perineuronal routes along the trigeminal or olfactory nerves.^10, 49, 50, 51, 52, 53^ Oxytocin levels in individuals with ASD are lower than those in typically developing individuals.^54, 55^ Therefore, sufficient amount of oxytocin is necessary to actually reach the brain in individuals with ASD to compensate for the lower oxytocin level.

In addition to dosage, we found that participants with the T-allele at rs6791619 showed stronger improvement of CGI-I score when dosage was lower (⩽21 IU per day). Although a recent meta-analysis revealed that rs6791619 is not solely associated with ASD occurrence,^21^ the SNP is considered a part of haplotype associated with impairment of core ASD symptoms.^37^ Wermter *et al.*^37^ found that 11 carriers of the T–G–T–T haplotype at rs237851–rs6791619–rs53576–rs237884 have more impairment than 89 noncarriers in social interaction and communication domains of the Autism Diagnostic Interview-Revised^56^ in individuals with ASD. There is a possibility that participants with T-allele at rs6791619 that showed stronger improvement in our clinical trial might be associated with the non-severe symptoms group reported by Wermter *et al.*^37^ However, since only one participant had T–G–T–T haplotype at rs237851–rs6791619–rs53576–rs237884 in the present trial, we cannot examine whether the efficacy was different between carriers of T–G–T–T haplotype at rs237851–rs6791619–rs53576–rs237884. In other words, 7 out of 8 participants (Figure 2), who did not have T alleles (that is, CC) at rs6791619, and who were not carriers of T–G–T–T haplotype at rs237851–rs6791619–rs53576–rs237884, showed lower efficacy of oxytocin administration. Therefore, we speculate a possibility that a SNP of rs6791619, rather than the haplotype at rs237851–rs6791619–rs53576–rs237884, might modulate responsibility of long-term oxytocin administration for males with ASD.

Collectively, we demonstrated that administration of >21 IU per day intranasal oxytocin is effective to alleviate comprehensive symptoms of ASD in males. Furthermore, when the dosage was ⩽21 IU per day, a SNP in *OXTR* (rs6791619) is associated with the efficacy of oxytocin administration. These findings should navigate future RCTs and contribute to establishing oxytocin administration as medical treatment in individuals with ASD.

### Safety

In the present trial, we did not observe any severe adverse events during a 12-week period of oxytocin administration. As this is the longest duration RCT of oxytocin in ASD to date (that is, previous RCTs lasted from 5 to 8 weeks), our data confirm the safety of long-term oxytocin treatment for use in future trials. Furthermore, oxytocin did not produce any abnormalities in the sex hormones, menstrual cycles or uterine peristaltic activity of the 13 female participants with ASD included in this RCT. Although oxytocin is known to induce uterine contractions,^57^ no other RCTs have evaluated the hormonal actions of long-term oxytocin administration in female participants. Thus, our results provide new evidence that long-term administration of oxytocin at a dose ⩽32 IU per day for 12 weeks does not lead to hormone-related abnormalities in non-pregnant females, which should encourage further RCTs on female participants with ASD.

### Limitation and further study

There are three limitations of the present pilot trial. First, although we provide the novel finding of dose-dependent efficacy with ⩽32 IU per day oxytocin and longer treatment duration than previous RCTs, we did not confirm the efficacy of higher dosage and time dependency in the double-blind phase. A rodent study showed negative finding such as deficit in partner preference behavior after long-term development treatment with lower doses of oxytocin.^58^ Therefore, further studies examining safety and efficacy at various dosages and administration periods are necessary. Second, because of smaller number of participants, we could not sufficiently examine the association between *OXTR* gene polymorphisms or sex and oxytocin efficacy. Further studies with larger sample size are warranted. Third, we found no significant changes in plasma oxytocin level. Non-extracted approaches may not have been appropriate as a methodology.^48, 59^

In conclusion, we showed dose- and genetic-dependent efficacy of oxytocin administration for comprehensive ASD-related symptoms in young males with high-functioning ASD. These findings are crucial for establishment of a therapeutic paradigm for oxytocin administration in individuals with ASD.

## Figures and Tables

**Table 1:** Participant demographics and baseline characteristics

	*Placebo group*			*Low-dose oxytocin group*			*High-dose oxytocin group*		
	*All (*n=*20)*	*Male (*n=*16)*	*Females (*n=*4)*	*All (*n=*20)*	*Male (*n=*18)*	*Females (*n=*2)*	*All (*n=*20)*	*Male (*n=*13)*	*Females (*n=*7)*
*Diagnosis,* n *(%)*									
Autistic disorder	18 (90%)	15 (94%)	3 (75%)	17 (85%)	15 (83%)	2 (100%)	19 (95%)	13 (100%)	6 (86%)
PDD-NOS	2 (10%)	1 (6%)	1 (25%)	3 (15%)	3 (17%)	0 (0%)	1 (5%)	0 (0%)	1 (14%)
									
Age (years), mean (s.d.)	24.9 (6.0)	25.3 (6.0)	23.3 (6.6)	23.1 (6.2)	22.9 (6.4)	24.5 (3.5)	24.8 (8.6)	27.8 (9.1)	19.0 (2.3)
Full-scale IQ, mean (s.d.)	98.5 (17.0)	100.8 (17.5)	90.3 (8.1)	99.2 (12.0)	99.9 (12.2)	93.5 (12.0)	102.2 (12.2)	103.9 (13.4)	99.0 (9.9)
AQ, mean (s.d.)	33.7 (5.8)	32.4 (5.6)	39.0 (2.9)	32.0 (5.2)	32.1 (5.4)	31.0 (2.8)	32.9 (5.2)	32.8 (6.0)	33.1 (3.6)
Number of participants that received psychotropic medications, *n* (%)	4 (20%)	2 (13%)	2 (50%)	5 (25%)	5 (28%)	0 (0%)	10 (50%)	6 (46%)	4 (57%)

Abbreviations: AQ, Autism-Spectrum Quotient; PDD-NOS, pervasive developmental disorder not otherwise specified (DSM-IV-TR).

There was no significant group difference in the ratio of participants with pervasive developmental disorder not otherwise specified among the three groups (*χ*^2^(2)=1.1, *P*=0.57).

**Table 2:** Summary of primary and secondary outcomes in the ITT population and subgroup population in male participants with good adherence

	*ITT population*				*Subgroup population (male participants with good adherence)*			
	*Placebo group (*n=*20)*	*Low-dose oxytocin group (*n=*20)*	*High-dose oxytocin group (*n=*20)*	*ANOVA interaction* P-*value (*n=*60)*	*Placebo group (*n=*14)*	*Low-dose oxytocin group (*n=*17)*	*High-dose oxytocin group (*n=*12)*	*ANOVA interaction* P-*value (*n=*43)*
*Primary outcome*								
Clinical Global Impression-Severity scale (CGI-S), mean (s.d.)								
week 0	6.0 (1.0)	5.8 (0.9)	5.9 (0.8)		5.8 (1.1)	5.9 (0.9)	5.8 (0.8)	
week 12	5.4 (1.1)	5.3 (0.9)	5.3 (1.3)	0.94	5.5 (1.2)	5.3 (1.0)	4.8 (1.2)	0.02
week 24	5.0 (1.3)	4.8 (1.0)	5.1 (1.4)		5.2 (1.3)	4.7 (1.0)	4.5 (1.1)	
Clinical Global Impression-Improvement scale (CGI-I),^a^ mean (s.d.)								
week 12	3.1 (1.1)	2.9 (1.2)	2.6 (1.2)	0.18	3.4 (0.9)	2.8 (1.2)	2.1 (1.0)	0.0011^b^
week 24	2.7 (1.2)	2.2 (1.1)	2.3 (1.4)		2.9 (1.0)	1.9 (1.0)	1.6 (0.8)	

*Interaction Rating Scale Advanced (IRSA), mean (s.d.)*								
week 0	256.0 (47.3)	262.4 (42.7)	272.6 (40.8)		251.3 (46.0)	259.8 (44.4)	283.4 (32.0)	
week 12	265.4 (55.3)	276.2 (44.2)	277.1 (50.4)	0.64	266.7 (43.4)	273.5 (46.0)	292.7 (37.0)	0.37
week 24	275.1 (49.6)	281.4 (46.0)	281.1 (40.6)		261.6 (46.4)	280.6 (47.4)	288.0 (35.5)	
								
*Secondary outcome*								
Aberrant Behavior Checklist I: Irritability/Agitation, mean (s.d.)								
week 0	10.7 (12.4)	8.8 (8.9)	4.7 (4.8)		6.3 (7.5)	9.3 (9.0)	3.4 (3.5)	
week 12	7.4 (9.5)	5.0 (5.9)	2.1 (4.5)	0.88	6.5 (9.6)	5.3 (6.0)	0.2 (0.7)	0.12
week 24	7.8 (10.9)	3.2 (4.8)	2.0 (4.3)		7.4 (10.7)	3.3 (4.9)	0.3 (0.7)	
Aberrant Behavior Checklist II: Lethargy/Social Withdrawal, mean (s.d.)								
week 0	13.3 (9.1)	19.5 (11.8)	16.8 (10.7)		11.3 (8.7)	20.8 (11.0)	17.7 (12.2)	
week 12	9.4 (10.5)	10.4 (9.4)	10.2 (10.9)	0.29	9.5 (11.2)	11.1 (9.3)	9.6 (12.1)	0.20
week 24	10.9 (12.7)	6.2 (6.2)	7.4 (8.1)		11.6 (13.7)	6.6 (6.2)	5.6 (5·9)	
Aberrant Behavior Checklist III: Stereotypic Behavior, mean (s.d.)								
week 0	3.1 (3.0)	4.0 (4.9)	2.5 (2.3)		3.0 (3.0)	4.3 (4.9)	2.8 (2.7)	
week 12	3.6 (4.4)	1.8 (3.2)	1.5 (2.3)	0.07	4.2 (4.7)	1.9 (3.3)	1.3 (1.7)	0.07
week 24	3.6 (4.0)	1.4 (2.9)	1.3 (2.6)		4.2 (4.2)	1.5 (3.0)	0.9 (1.5)	
Aberrant Behavior Checklist IV: Hyperactivity/Noncompliance, mean (s.d.)								
week 0	9.0 (8.2)	8.3 (8.1)	4.9 (5.5)		6.8 (7.2)	8.9 (8.1)	4.7 (5.5)	
week 12	6.2 (8.3)	3.7 (5.2)	2.6 (5.6)	0.92	5.9 (8.9)	3.9 (5.2)	1.3 (3.0)	0.40
week 24	7.3 (10.4)	2.8 (4.3)	2.4 (5.4)		7.4 (11.0)	2.9 (4.3)	0.8 (1.8)	
Aberrant Behavior Checklist V: Inappropriate Speech, mean (s.d.)								
week 0	3.1 (3.6)	1.6 (2.6)	1.2 (2.1)		2.5 (3.1)	1.7 (2.7)	0.3 (0.5)	
week 12	2.3 (2.8)	1.1 (2.4)	0.6 (1.5)	0.85	2.3 (3.0)	1.2 (2.5)	0.2 (0.7)	0.89
week 24	3.0 (3.5)	0.8 (1.9)	0.7 (1.3)		3.1 (3.6)	0.8 (1.9)	0.1 (0.4)	
Zung Self-Rating Depression Scale, mean (s.d.)								
week 0	48.3 (8.8)	49.0 (8.1)	50.1 (9.0)		47.8 (7.1)	48.8 (8.3)	47.5 (10.9)	
week 12	48.4 (8.0)	47.4 (10.6)	47.2 (9.8)	0.24	48.2 (8.5)	46.8 (10.6)	44.5 (10.6)	0.24
week 24	47.5 (8.2)	44.6 (11.0)	46.9 (10.6)		47.2 (8.1)	44.1 (11.2)	44.6 (11.0)	
State Anxiety Scale of State-Trait Anxiety Inventory, mean (s.d.)								
week 0	53.0 (8.9)	52.0 (10.2)	52.0 (12.7)		51.7 (8.0)	52.2 (10.5)	47.9 (14.2)	
week 12	49.8 (10.1)	50.2 (13.4)	48.3 (13.6)	0.95	47.9 (10.1)	49.9 (13.7)	44.5 (13.9)	0.94
week 24	52.0 (12.7)	48.4 (11.7)	49.5 (13.0)		49.0 (12.2)	47.9 (11.8)	45.6 (12.4)	
The 20-item Toronto Alexithymia Scale, mean (s.d.)								
week 0	62.4 (6.9)	58.1 (10.5)	61.4 (14.4)		61.3 (7.0)	57.9 (10.8)	61.2 (16.4)	
week 12	60.5 (13.1)	58.2 (13.0)	57.2 (14.8)	0.47	56.8 (10.5)	58.7 (13.3)	56.7 (18.1)	0.99
week 24	60.2 (13.8)	56.0 (17.0)	60.6 (13.9)		56.4 (11.3)	55.8 (17.6)	59.3 (18.0)	
								
*Monitoring item*								
Plasma oxytocin concentration (pg ml^−1^), mean (s.d.)								
week 0	226.6 (125.7)	251.2 (169.0)	285.0 (198.3)		208.6 (96.1)	262.9 (173.4)	256.4 (195.6)	
week 12	231.7 (104.9)	221.7 (109.5)	265.6 (176.4)	0.61	216.3 (106·0)	228.9 (112.6)	220.6 (124.1)	0.46
week 24	231.6 (128.1)	213.2 (142.0)	237.7 (167.1)		192.5 (74.9)	230.7 (136.7)	205.2 (127.5)	

Abbreviations: ANOVA, analysis of variance; ITT, intention-to-treat.

ANOVA indicates a two-way ANOVA of each outcome with group (high-dose oxytocin group and placebo group) and time (weeks 0 and 12) as factors in the double-blind phase, unless otherwise indicated.

a For only CGI-I, *t*-test was performed for the high-dose oxytocin group and placebo groups at week 12 (two-sided).

b *P*<0.004 (0.05/11).

**Table 3:** Adverse events

	*During double-blind phase (12 weeks from week 0 to 11)*			*During open-label and follow-up phase (20 weeks from week 12 to 31)*		
	*Placebo group (*n=*20)* (M=16, F=4)	*Low-dose oxytocin group (*n=*20)* (M=18, F=2)	*High-dose oxytocin group (*n=*20)* (M=13, F=7)	*Placebo group (*n=*18)* (M=14, F=4)	*Low-dose oxytocin group (*n=*18)* (M=17, F=1)	*High-dose oxytocin group (*n=*19)* (M=12, F=7)
Total adverse even*ts, n (%)*	6 (30)	5 (25)	3 (15)	5 (28)	1 (6)	7 (33)
Patients reporting one or more adverse events, *n* (%)	4 (20)	4 (20)	3 (15)	5 (28)	1 (6)	5 (26)
						
*Specific adverse events,* n *(%)*						
Somnolence	1 (5)	1 (5)	1 (5)	1 (6)	1 (6)	2 (11)
Feeling of floating	1 (5)	0	0	0	0	1 (5)
Palpitation	1 (5)	0	0	0	0	0
Nausea	0	0	0	1 (6)	0	0
Hypersensitive olfactory stimuli	0	1 (5)	0	0	0	0
Itching of the nose	1 (5)	0	0	0	0	0
Heat sensation of the glabella	0	1 (5)	0	0	0	0
Acoustic hyperesthesia	0	0	0	0	0	0
Abnormal sensation of lower limb	0	0	0	0	0	1 (5)
Decreased activity	0	0	1 (5)	0	0	1 (5)
Worsened obsessive behavior	1 (5)	1 (5)	0	1 (5)	0	1 (5)
Irritability	1 (5)	0	0	1 (5)	0	0
Hyperthymia	0	0	0	0	0	1 (5)
Excessive contact with acquaintances	0	1 (5)	1 (5)	0	0	0
Playful attitude	0	0	0	1 (6)	0	0
						
*Specific adverse events (female participants)*						
Abnormal change of sex hormone level	0	0	0	0	0	0
Menstrual problem	0	0	0	0	0	0
Galactorrhea	0	0	0	0	0	0
Abnormal uterine peristalsis	0	0	0	0	0	0

Abbreviations: ANOVA, analysis of variance; F, female participants; M, male participants; NA, not available.

During the double-blind phase, one-way ANOVA showed no significant differences in the number of patients who experienced one or more adverse events among the groups (*P*>0.05).
