# Supplementary material for: Oxytocin efficacy is modulated by dosage and oxytocin receptor genotype in young adults with high-functioning autism: a 24-week randomized clinical trial
Source: Transl Psychiatry. 2016 Aug 23;6(8):e872–. doi: 10.1038/tp.2016.152 (PMC5022092; doi:10.1038/tp.2016.152)
Supplement: Supplementary Information [file tp2016152x1.docx]

**Supplementary Table 1.**

|  | **Supplementary Table 1.** Genotype frequencies of 24 single-nucleotide polymorphisms (SNPs) of the oxytocin receptor (*OXTR*) in the study sample | | | | | | | | | | |  |
| --- | --- | --- | --- | --- | --- | --- | --- | --- | --- | --- | --- | --- |
|  |  |  | *OXTR* | ChAomal |  |  | Minor | MAF | Frequency† | | |  |
|  |  | No. | SNP ID | Position (bp) | Location | Alleles | Allele | (JPT) | 1/1 | 1/2 | 2/2 |  |
|  | * | 1 | rs237851 | 8793037 | 5' region | A/G | G | 0.262 | 22 | 6 | 0 |  |
|  | * | 2 | rs6791619 | 8788186 | 5' region | C/T | T | 0.308 | 11 | 12 | 5 |  |
|  |  | 3 | rs2270465 | 8775290 | 5' region | C/G | C | 0.5 | 7 | 19 | 2 |  |
|  | * | 4 | rs4643699 | 8771307 | 5' region | A/T | A | 0.095 | 20 | 8 | 0 |  |
|  |  | 5 | rs2301261 | 8769210 | 5' region, exon 1 | A/G | A | 0.122 | 24 | 4 | 0 |  |
|  | * | 6 | rs4686302 | 8767536 | Exon 3 | C/T | T | 0.198 | 11 | 12 | 5 |  |
|  | * | 7 | rs237897 | 8766599 | Intron 3 | A/G | A | 0.681 | 7 | 15 | 6 |  |
|  | * | 8 | rs2268495 | 8765849 | Intron 3 | A/G | A | 0.198 | 12 | 11 | 5 |  |
|  | * | 9 | rs53576 | 8762685 | Intron 3 | A/G | A | NA | 6 | 17 | 5 |  |
|  | * | 10 | rs11131149 | 8761165 | Intron 3 | A/G | A | 0.169 | 21 | 5 | 2 |  |
|  | * | 11 | rs2254295 | 8760606 | Intron 3 | C/T | C | 0.285 | 18 | 8 | 2 |  |
|  | * | 12 | rs2254298 | 8760542 | Intron 3 | A/G | A | 0.311 | 18 | 8 | 2 |  |
|  |  | 13 | rs2268494 | 8760360 | Intron 3 | A/T | A | 0.062 | 2 | 26 | 0 |  |
|  | * | 14 | rs2268493 | 8759154 | Intron 3 | C/T | C | 0.077 | 21 | 7 | 0 |  |
|  | * | 15 | rs2268491 | 8758712 | Intron 3 | C/T | T | 0.285 | 18 | 8 | 2 |  |
|  | * | 16 | rs4686301 | 8756900 | Intron 3 | C/T | T | 0.203 | 19 | 7 | 2 |  |
|  | * | 17 | rs9840864 | 8756791 | Intron 3 | C/G | C | 0.738 | 9 | 16 | 3 |  |
|  | * | 18 | rs918316 | 8756495 | Intron 3 | C/T | C | 0.238 | 14 | 14 | 0 |  |
|  | * | 19 | rs2268490 | 8755399 | Intron 3 | C/T | T | 0.442 | 13 | 10 | 5 |  |
|  | * | 20 | rs237887 | 8755356 | Intron 3 | A/G | G | 0.564 | 10 | 11 | 7 |  |
|  | * | 21 | rs11706648 | 8754861 | Intron 3 | A/C | C | 0.279 | 12 | 13 | 3 |  |
|  | * | 22 | rs237885 | 8753857 | Intron 3 | G/T | G | 0.244 | 14 | 12 | 2 |  |
|  |  | 23 | rs1042778 | 8752859 | 3'UTR, exon4 | C/G/T | T | 0.093 | 22 | 6 | 0 |  |
|  |  | 24 | rs237884 | 8751899 | 3'UTR, exon4 | A/G | G | 0 | 28 | 0 | 0 |  |
|  | Abbreviation: *OXTR*, oxytocin receptor; SNP, single-nucleotide polymorphism; MAF (JPT), minor allele frequency in Japanese population in the HapMap project; NA, not available. *, 19 SNPs that showed Hardy Weinberg Equilibrium (HWE) in the dataset (*P*>0.05) or minor allele frequencies above 5% in the Japanese population. †Genotypes, major and minor alleles are denoted by 1 and 2, respectively. | | | | | | | | | | |  |

**Supplementary Information: 1. Gazefinder**

Gaze pattern is considered to be associated with social behaviors^1, 2^ and abnormalities in gaze pattern are frequently found in individuals with autism spectrum disorders (ASD).^3-7^ Single-dose oxytocin administration increases the amount of time individuals with ASD gaze at the eye region of the human face.^8, 9^ Therefore, oxytocin administration might facilitate social behaviors in individuals with ASD through modulation of gaze behavior. However, it is unknown whether improvement of gaze abnormalities also occurs after long-term oxytocin administration. To examine whether gaze abnormality is improved by long-term oxytocin administration, we measured fixation time on social stimuli such as the eye region of human faces or biological motion using an eye-tracking system in individuals with ASD.

The outcome was measured after April 2013 for participants gave additional informed consent (n=23). Therefore, we acquired data from 18 male participants in the subgroup population. Four participants were excluded from data analysis due to low available percentage of fixation time (<80%). Therefore, data of 14 participants (4 males in the high-dose oxytocin group, 5 males in the low-dose oxytocin group, and 5 males and in the placebo group) were analyzed. We utilized Gazefinder (JVCKENWOOD, Yokohama, Japan)^6^ for stimulus presentation and data correction. The Gazefinder is an eye-tracking system that was developed to measure the unique gaze fixation patterns characteristic of ASD. Although the Gazefinder was originally developed to measure gaze patterns in children with ASD, we recently confirmed that the Gazefinder can also be used to measure gaze patterns in young adults with ASD.^10^ Eight movies including human faces, biological motion, and people and geometry were presented (approximately 2 min, Table S1). We calculated the percentage of fixation time for the eye region in human face movies (still image, eye blinking, mouth moving, silent, and talking stimuli), upright biological motion images for biological motion movies (an upright human dancing to a song), people for movies of people and geometric shapes [same size] (the movies of people and geometric shapes were presented at the same time and at the same size) and the geometric shapes for movies of people and geometric shapes [small window] (the movies of geometric shapes were depicted in small-frame images superimposed on the movies of people). Similar to the approach for the primary and secondary outcomes, we utilized two-step analysis with a gatekeeping strategy for data analysis.

In the first step of analysis, two-way ANOVA with groups (high-dose/placebo) and time (weeks 0/12) revealed significant interaction in biological motion (*P*=0.002, Cohen’s d=3.56). Although other stimuli did not show significant interaction, they showed large effect size^11^ (Table S1). In the second step analysis, a result of a two-way ANOVA with groups (high-dose/placebo) and time (weeks 0/12) for the low-dose oxytocin and placebo groups in biological motion was not significant (*P*=0.14).

Previous study also found that individuals with ASD did not show a preference for biological motion motion.^5, 12^ Thus, our results suggest that high-dose, long-term oxytocin administration improves abnormality in gaze behavior. The improvement of fixation time for biological motion was similar to improvement in Clinical Global Impression-Improvement (CGI-I) scores, and both measures showed improvement in the high-dose but not in the low-dose group. Therefore, it is reasonable that changes in gaze pattern contribute to the improvement of general symptoms measured by the CGI-I.

**Supplementary Table 2**

|  | **Supplementary Table 2.** Summary of the percentages of fixation times and effect size in Gazefinder in the subgroup of male participants with good adherence | | | | | | | |  |
| --- | --- | --- | --- | --- | --- | --- | --- | --- | --- |
|  |  |  |  | *Subgroup population* | | | | |  |
|  |  |  |  | *(male participants with good adherence)* | | | | |  |
|  |  |  |  |  | low-dose | high-dose | ANOVA |  |  |
|  |  |  |  | placebo | oxytocin | oxytocin | Interaction |  |  |
|  |  |  |  | group | group | group | p value | Effect size |  |
|  |  |  |  | (n=5) | (n=5) | (n=4) | (n=14) | Cohen's d |  |
|  | *Human face (AOI: eye region)* | | | | | | |  |  |
|  |  | Still, mean (s.d.) | | | | | |  |  |
|  |  |  | week 0 | 71.3 (31.7) | 67.4 (32.6) | 61.2 (22.7) |  |  |  |
|  |  |  | week 12 | 59.7 (30.8) | 78.9 (19.5) | 75.9 (27.4) | 0.09 | 1.51* |  |
|  |  | Blinking, mean (s.d.) | | | | | |  |  |
|  |  |  | week 0 | 65.8 (26.7) | 40.9 (31.3) | 58.8 (29.4) |  |  |  |
|  |  |  | week 12 | 57.8 (36.3) | 65.7 (26.0) | 78.9 (33.4) | 0.11 | 1.38* |  |
|  |  | Mouth moving, mean (s.d.) | | | | | |  |  |
|  |  |  | week 0 | 42.8 (31.8) | 24.2 (19.6) | 49.4 (36.8) |  |  |  |
|  |  |  | week 12 | 42.8 (17.6) | 39.3 (30.2) | 58.1 (32.8) | 0.56 | 0.46 |  |
|  |  | Silent, mean (s.d.) | | | | | |  |  |
|  |  |  | week 0 | 43.3 (51.9) | 24.3 (43.3) | 45.5 (36.8) |  |  |  |
|  |  |  | week 12 | 55.9 (34.5) | 35.7 (48.2) | 48.2 (52.1) | 0.75 | 0.25 |  |
|  |  | Talking, mean (s.d.) | | | | | |  |  |
|  |  |  | week 0 | 44.1 (44.8) | 27.0 (28.1) | 43.8 (44.4) |  |  |  |
|  |  |  | week 12 | 52.3 (35.1) | 35.2 (38.4) | 47.4 (38.5) | 0.86 | 0.14 |  |
|  | *Biological motion (AOI: Upright), mean (s.d.)* | | | | | | |  |  |
|  |  |  | week 0 | 54.7 (12.7) | 47.0 (17.8) | 44.5 (10.6) |  |  |  |
|  |  |  | week 12 | 39.6 (9.6) | 54.4 (22.6) | 53.1 (3.7) | 0.002† | 3.56* |  |
|  | *People and geometry [same size], (AOI: People), mean (s.d.)* | | | | | | |  |  |
|  |  |  | week 0 | 57.6 (22.4) | 38.1 (22.0) | 57.9 (19.1) |  |  |  |
|  |  |  | week 12 | 52.2 (18.4) | 38.5 (27.6) | 59.6 (15.6) | 0.24 | 0.98* |  |
|  | *People and geometry [small window], (AOI: Geometry), mean (s.d.)* | | | | | | |  |  |
|  |  |  | week 0 | 32.1 (27.0) | 32.0 (26.5) | 38.4 (29.2) |  |  |  |
|  |  |  | week 12 | 36.1 (30.7) | 30.4 (25.0) | 28.9 (13.5) | 0.21 | 1.04* |  |
|  | ANOVA indicates a two-way ANOVA of each outcome with group (high-dose oxytocin group and placebo group) and time (weeks 0 and 12) as factors in the double-blind phase. Effect sizes reflect differences between the high-dose oxytocin group and the placebo group during the double-blind phase (week 0 to week 12). †*P*<0.006 (0.05/8). *large effect size (>0.80).^10^ | | | | | | | |  |
|  |  |  |  |  |  |  |  |  |  |
|  |  |  |  |  |  |  |  |  |  |

**1-References**

1. Emery NJ. The eyes have it: the neuroethology, function and evolution of social gaze. *Neuroscience and Biobehavioral Reviews* 2000; **24:** 581-604.

2. Powell KL, Roberts G, Nettle D. Eye images increase charitable donations: evidence from an opportunistic field experiment in a supermarket. *Ethology* 2012; **118:** 1096–1101.

3. Dalton KM, Nacewicz BM, Johnstone T, Schaefer HS, Gernsbacher MA, Goldsmith HH *et al.* Gaze fixation and the neural circuitry of face processing in autism. *Nat Neurosci* 2005; **8**(4)**:** 519-526.

4. Corden B, Chilvers R, Skuse D. Avoidance of emotionally arousing stimuli predicts social-perceptual impairment in Asperger's syndrome. *Neuropsychologia* 2008; **46**(1)**:** 137-147.

5. Klin A, Lin DJ, Gorrindo P, Ramsay G, Jones W. Two-year-olds with autism orient to non-social contingencies rather than biological motion. *Nature* 2009; **459:** 257-261.

6. Fujisawa TX, Tanaka S, Saito DN, Kosaka H, Tomoda A. Visual attention for social information and salivary oxytocin levels in preschool children with autism spectrum disorders: an eye-tracking study. *Front Neurosci* 2014; **8:** 295.

7. Shic F, Bradshaw J, Klin A, Scassellati B, Chawarska K. Limited activity monitoring in toddlers with autism spectrum disorder. *Brain Res* 2011; **1380:** 246-254.

8. Andari E, Duhamel JR, Zalla T, Herbrecht E, Leboyer M, Sirigu A. Promoting social behavior with oxytocin in high-functioning autism spectrum disorders. *Proc Natl Acad Sci U S A* 2010; **107:** 4389-4394.

9. Auyeung B, Lombardo MV, Heinrichs M, Chakrabarti B, Sule A, Deakin JB, et al. Oxytocin increases eye contact during a real-time, naturalistic social interaction in males with and without autism. *Transl Psychiatry* 2015; **5**: e507.

10. Fujioka T, Inohara K, Okamoto Y, Masuya Y, Ishitobi M, Saito DN *et al.* Gazefinder as a clinical supplementary tool for discriminating between autism spectrum disorder and typical development in male adolescents and adults. *Mol Autism* 2016; **7:** 19.

11. Cohen J: A power primer. *Psychol Bull* 1992; **112:** 155-159.

12. Falck-Ytter T, Rehnberg E, Bölte S. Lack of visual orienting to biological motion and audiovisual synchrony in 3-year-olds with autism. *PLoS One* 2013; **8**(7)**:** e68816.

**Supplementary Information: 2. Resting state functional MRI**

Single-dose and long-term oxytocin administration showed improvement of altered brain function in individuals with ASD in previous studies.^1–5^ For instance, Watanabe et al.^5^ found changes of brain activity not only during the performance of specific tasks, but also in the resting state. In this trial, we performed resting state functional MRI (rs-fMRI) to confirm whether long-term oxytocin administration changes resting-state brain function in individuals with ASD.

The outcome was measured after July 2013 for participants who gave additional informed consent (n=18). Therefore, we acquired data from 14 male participants in the subgroup population. Three participants were excluded from data analysis due to excessive head movement (>3 mm). Because the remaining participants were 1 male in the high-dose oxytocin group, 5 males in the low-dose oxytocin group, and 5 males in the placebo group, we only compared brain activity between the low-dose and placebo groups. Using a 3T MR scanner (Discovery MR 750; General Electric Medical Systems, Milwaukee, WI), we acquired images using a T2*-weighted gradient-echo echo-planar imaging sequence^6^. The data were analyzed using SPM8, a data processing assistant, and rs-fMRI software (DPARSF). The fractional amplitude of low-frequency fluctuation (fALFF) map was calculated as the ratio of the power spectrum of the band ranging from 0.01 to 0.08 Hz to that of the entire frequency range. The fALFF was then standardized voxel-wise into a subject-level Z-score fALFF map, which can reduce influence of head motion and thus provide information about the power of spontaneous low frequency fluctuations relative to all frequencies.^7^ For group comparisons, each image pertaining to z values reflecting fALFF was subjected to two-way repeated measures ANOVA with groups (low-dose/placebo) and time (weeks 0/12) as factors, using a threshold set at p<0.005 uncorrected for height and k>10.

Compared to values in the placebo group, fALFF (week 12 > week 0) was increased in the left insular cortex and left temporal pole, and decreased in the right insular cortex, bilateral supplementary motor areas, left precentral gyrus, and right postcentral gyrus for the low-dose group (Table S2).

We also found a significant difference in fALFF between the low-dose oxytocin and placebo groups. The present results showed that in addition to influencing various brain regions, long-term oxytocin administration may also result in area-specific increases and decreases in activity. The regions with increased fALFF such as the left insular cortex and left temporal pole are involved in speech and sensory processing^8^, responses to social scenes and facial stimuli,^9^ and emotional reactions to social stimuli.^10^ Although no significant difference was observed in behaviors between the oxytocin and placebo groups, oxytocin administration changes activity of brain regions that are putatively involved in the core symptoms of ASD.

**Supplementary Table 3**

|  | **Supplementary Table 3.** Brain regions showing significant interaction for the fractional amplitude of low-frequency fluctuations (fALFF) between group (low-dose oxytocin group and placebo group) and time (weeks 0 and 12) | | | | | | | | | |  |
| --- | --- | --- | --- | --- | --- | --- | --- | --- | --- | --- | --- |
|  | Brain regions | | BA | MNI coordinates | | | *Z*-score | Cluster size | | Uncorrected |  |
|  |  |  |  | x | y | z |  | *k* (voxels) | | *p* |  |
|  | Increased fALFF (week 12 > week 0) in low-dose oxytocin group | | | | | | | | | |  |
|  |  | Lt. Insular cortex | 13 | -36 | 10 | -14 | 3.05 | 18 | 0.001 | |  |
|  |  | Lt. Temporal pole | - | -42 | 12 | -24 | 2.99 | 12 | 0.001 | |  |
|  | Decreased fALFF (week 0 > week 12) in low-dose oxytocin group | | | | | | | | | |  |
|  |  | Rt. Supplementary motor area | - | 12 | -10 | 62 | 4.18 | 21 | <0.001 | |  |
|  |  | Lt. Supplementary motor area | - | -6 | -10 | 60 | 3.67 | 13 | <0.001 | |  |
|  |  | Lt. Precentral gyrus | 6 | -38 | -10 | 46 | 3.63 | 74 | <0.001 | |  |
|  |  | Rt. Insular cortex | - | 34 | -6 | 14 | 3.30 | 18 | <0.001 | |  |
|  |  | Rt. Postcentral gyrus | 40 | 64 | -24 | 18 | 2.95 | 14 | 0.002 | |  |
|  |  | Lt. Precentral gyrus | - | -52 | -8 | 44 | 2.80 | 13 | 0.003 | |  |
|  | The statistical threshold for contrasts was p<0.005 uncorrected for height and cluster k>10 for extent.  Abbreviation: Lt, left; Rt, right. | | | | | | | | | |  |
|  |  |  |  |  |  |  |  |  |  |  |  |

**2-References**

1 Watanabe T, Abe O, Kuwabara H, Yahata N, Takano Y, Iwashiro N *et al.* Mitigation of Sociocommunicational Deficits of Autism Through Oxytocin-Induced Recovery of Medial Prefrontal Activity. *JAMA Psychiatry* 2013; **7**1: 166–175.

2 Domes G, Kumbier E, Heinrichs M, Herpertz SC. Oxytocin promotes facial emotion recognition and amygdala reactivity in adults with asperger syndrome. *Neuropsychopharmacology* 2014; **39:** 698–706.

3 Domes G, Heinrichs M, Kumbier E, Grossmann A, Hauenstein K, Herpertz SC. Effects of intranasal oxytocin on the neural basis of face processing in autism spectrum disorder. *Biol Psychiatry* 2013; **74:** 164–171.

4 Aoki Y, Yahata N, Watanabe T, Takano Y, Kawakubo Y, Kuwabara H *et al.* Oxytocin improves behavioural and neural deficits in inferring others’ social emotions in autism. *Brain* 2014; **137:** 3073–3086.

5 Watanabe T, Kuroda M, Kuwabara H, Aoki Y, Iwashiro N, Tatsunobu N *et al.* Clinical and neural effects of six-week administration of oxytocin on core symptoms of autism. *Brain* 2015; **138:** 3400–3412.

6 Jung M, Kosaka H, Saito DN, Ishitobi M, Morita T, Inohara K *et al.* Default mode network in young male adults with autism spectrum disorder: relationship with autism spectrum traits. *Mol Autism* 2014; **5:** 35.

7 Jung M, Mody M, Saito DN, Tomoda A, Okazawa H, Wada Y *et al.* Sex Differences in the Default Mode Network with Regard to Autism Spectrum Traits: A Resting State fMRI Study. *PLoS One* 2015; **10:** e0143126.

8 Kurth F, Zilles K, Fox PT, Laird AR, Eickhoff SB. A link between the systems: functional differentiation and integration within the human insula revealed by meta-analysis. *Brain Struct Funct* 2010; **5-6:** 1–16.

9 Wigton R, Radua J, Allen P, Averbeck B, Meyer-Lindenberg A, McGuire P *et al.* Neurophysiological effects of acute oxytocin administration: systematic review and meta-analysis of placebo-controlled imaging studies. *J Psychiatry Neurosci* 2015; **40:** E1–E22.

10 Olson IR, Plotzker A, Ezzyat Y. The Enigmatic temporal pole: a review of findings on social and emotional processing. *Brain* 2007; **130:** 1718–1731.
